# Landscape of Aeroallergen Sensitization in Asia

**DOI:** 10.1111/cea.70137

**Published:** 2025-08-18

**Authors:** Xiaoling Yin, Xiangling Zhang, Feng Xu, Hao Xiao, Juan Meng, Philip H. Li

**Affiliations:** ^1^ Allergy Center West China Hospital, Sichuan University Sichuan China; ^2^ Department of Otolaryngology‐Head and Neck Surgery West China Hospital, Sichuan University Sichuan China; ^3^ Division of Rheumatology and Clinical Immunology, Department of Medicine Queen Mary Hospital, University of Hong Kong Hong Kong SAR China; ^4^ Division of Rheumatology and Clinical Immunology, Department of Medicine University of Hong Kong‐Shenzhen Hospital Guangdong China

**Keywords:** aeroallergen, allergen immunotherapy, allergic sensitization, Asia

## Abstract

Allergic diseases caused by aeroallergens are emerging as a critical public health issue across Asia. Diverse climatic and geographic conditions play a significant role in shaping regional variation in aeroallergy sensitisation patterns across Asia. House dust mites (HDMs) remain the predominant indoor allergens, while pollen sensitisation varies substantially based on regional flora. Rapid urbanisation, evolving lifestyles, increasing air pollution, and genetic susceptibility further influence allergen exposure and sensitisation profiles. Although pharmacological and biologic therapies have advanced, allergen‐specific immunotherapy (AIT) remains the only disease‐modifying therapeutic strategy. This review synthesises current evidence on aeroallergen sensitisation patterns across Asia, elucidates key influencing factors across various Asian regions, and discusses current prevention and treatment strategies, emphasising the role and challenges of AIT. Improved understanding of these regional allergenic profiles and management practices is essential for developing tailored clinical guidelines and public health interventions.

## Graphical abstract

In Asia, aeroallergen sensitisation is driven by climate, geography, pollution, and demographic shifts, dominated by house dust mites indoors and varying pollen sensitisation. Allergen immunotherapy remains underutilised, especially in low‐ and middle‐income countries, due to standardisation gaps and limited access to AIT products, hindering equitable access to treatment.

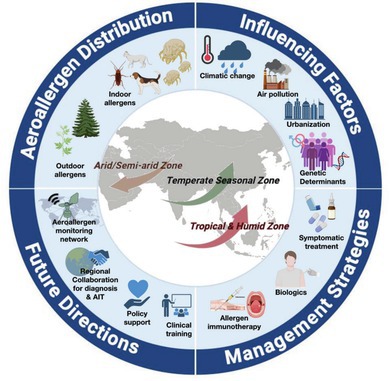

**Summary**

- Aeroallergen sensitisation in Asia is influenced by climatic conditions, geographic diversity, pollution, and demographic shifts.
- House dust mites are the predominant indoor allergen, while pollen sensitisation varies geographically.
- Allergen immunotherapy remains underutilised, especially in low‐ and middle‐income Asian countries.
- Lack of standardisation and limited availability of AIT products remain significant barriers to equitable access to treatment.

## 1 Introduction

In recent years, allergic diseases—including allergic asthma (AA), allergic rhinitis (AR)—have emerged as a growing public health challenge across Asia. Epidemiological studies report high and increasing prevalence rates: AR affects nearly one‐third of the population in some Asian countries (e.g., 27% in South Korea, 32% in the United Arab Emirates [1]), and AA impacts tens of millions, with South Asia alone accounting for nearly 40 million cases as of 2019 [2]. In contrast to the plateauing trends observed in many Western nations, the burden of allergic diseases in Asia continues to rise, particularly in urban centres [3].

Sensitization to aeroallergens such as house dust mites (HDMs), moulds, pollens, and animal dander plays a central role in the onset and progression of allergic diseases. Asia's climatic and ecological diversity, from tropical monsoon to temperate climates, contributes to considerable heterogeneity in aeroallergen exposure across the region. While HDMs sensitization is widespread, it demonstrates marked regional variation, ranging from 10% to 20% in parts of India and Vietnam to over 85%–90% in Singapore and southern China [4, 5, 6]. Seasonal pollen surges are more pronounced in temperate areas [7], especially *Cryptomeria japonica* (Japanese cedar) in Japan and *Artemisia vulgaris* (mugwort) in northern China, particularly Inner Mongolia [8, 9], imposing a substantial burden on public health.

In addition to natural factors, rapid industrialisation and urbanisation across Asia have exacerbated allergic disease prevalence [10, 11, 12, 13]. Air pollution (e.g., PM_2.5_, PM_10_, NO_2_) alters allergen structures, enhances allergen potency, and intensifies allergic airway inflammation. For example, in Japan, the expansion of cedar forests combined with air pollution and climate warming has markedly increased airborne cedar pollen levels, with sensitisation rates reaching 60% among young adults [14]. Demographic changes such as sex and age also influence sensitisation patterns, with generally higher rates observed in males and younger children, with sensitisation to certain allergens increasing with age [15, 16, 17, 18].

In this review, we aim to synthesise current evidence on aeroallergen sensitisation across Asia. Allergen sensitisation has been assessed primarily through skin prick tests (SPT) and/or specific serum IgE testing. We integrate findings from across the region to delineate the spectrum of common aeroallergens and their sensitisation rates. Environmental influences (such as climate, air pollution, and degree of urbanisation) and population demographics (age, sex, atopic predisposition) are explored as potential contributors to regional variability in sensitisation patterns. Furthermore, we discuss the clinical implications for management, especially the use of specific allergen immunotherapy (AIT) in different Asia countries. By delineating Asia's unique sensitisation landscape, this review aims to inform region‐specific prevention strategies, guide clinical management practices—including the wider implementation of AIT—and identify future research priorities in allergy diagnostics and treatment.

## 2 Aeroallergen Patterns in Asia

### 2.1 House Dust Mites: The Dominant Asian Allergen

**FIGURE 1:**
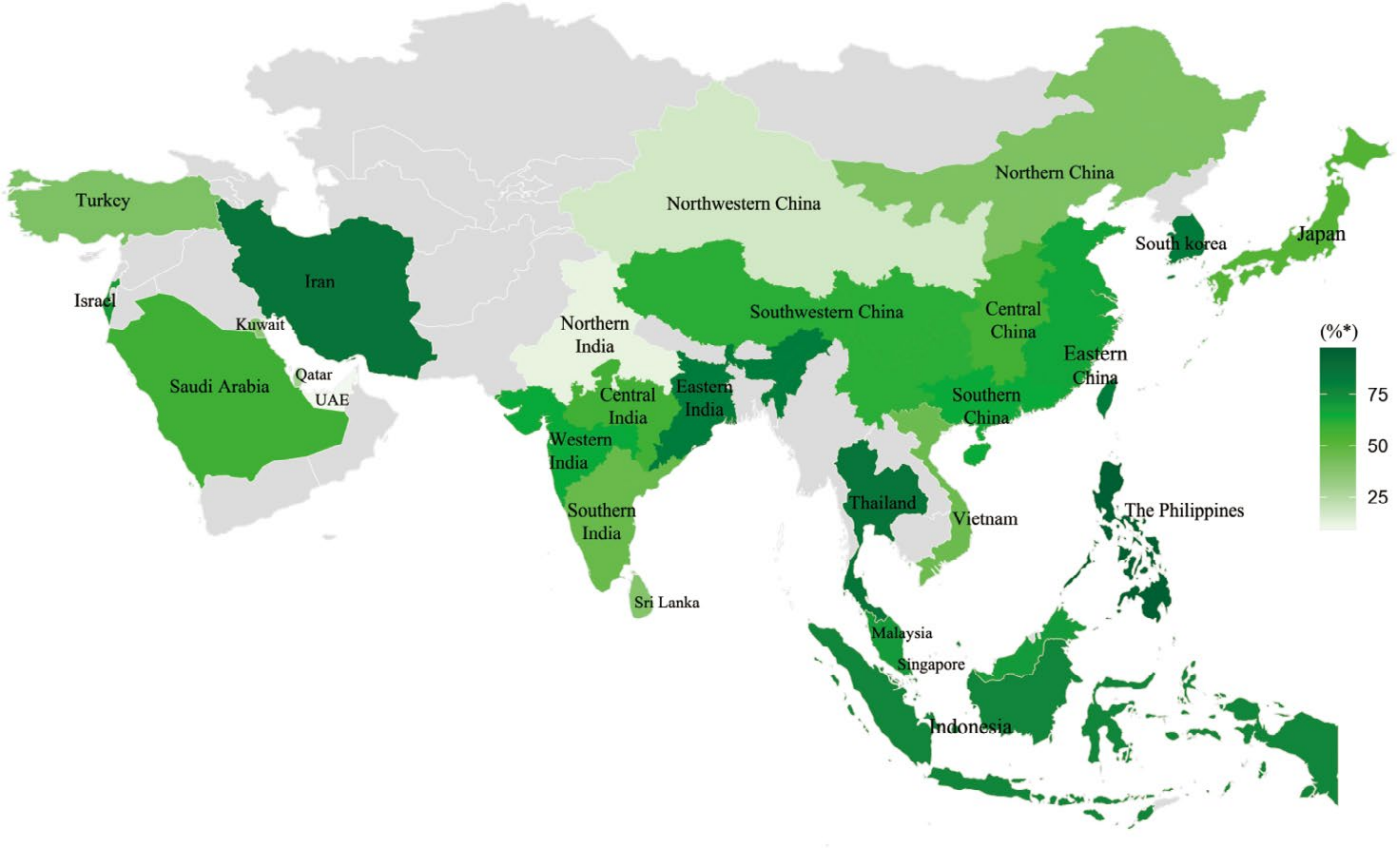
Geographic heatmap of house dust mite sensitisation rates across Asia. *Heatmap showing sensitisation rates (%) to HDMs (*D. pteronyssinus*, *D. farinae*, *B. tropicalis* ) based on SPT or sIgE results from Asian studies.

HDMs constitute the primary source of indoor aeroallergens throughout Asia. Sensitisation patterns exhibit considerable geographic variation, predominantly driven by climatic factors, indoor humidity levels, and socio‐environmental conditions. In temperate East Asia, *Dermatophagoides pteronyssinus* (*D. pteronyssinus*) and *Dermatophagoides farina* (*D. farinae*) are the dominant species, while in the tropical climates of Southeast Asia, high sensitisation rates are observed for both *Blomia tropicalis* ( *B. tropicalis* ) and *D. pteronyssinus* [19, 20, 21]. In South Asia, regional variation in humidity levels contributes to the coexistence of multiple HDM species in coastal zones, while arid inland areas are dominated by *D. farinae* due to its desiccation resistance [22, 23, 24]. To illustrate regional distribution, we compiled sensitisation rates to *D. pteronyssinus*, *D. farinae*, and *B. tropicalis* reported across Asian countries over the past decade. Given the limited availability of recent studies in certain regions, earlier publications (≥ 10 years) have been included to ensure completeness (Figure 1, Created with R version 4.4.3).

In East Asia, temperate and subtropical climates favour the proliferation of *D. pteronyssinus* and *D. farinae*, with sensitization rates ranging from 20% to 80% among individuals with AR or AA [25, 26, 27, 28]. In northern China (e.g., Beijing), *D. farinae* is the dominant species, while *D. pteronyssinus* and *B. tropicalis* are more prevalent in southern regions such as Guangdong and Taiwan, with the latter reaching 63.8% in allergic children [25, 29, 30, 31]. In Southeast Asia, the consistently humid tropical climate supports the proliferation of both *D. pteronyssinus* and *B. tropicalis* . In Malaysia's Klang Valley, *B. tropicalis* has become the most abundant mite species (~8900 mites/g dust) [32], reflecting a notable species shift from *D. pteronyssinus* to *B. tropicalis* since the 1990s, likely due to the shorter developmental cycle of *B. tropicalis* [33]. Singaporean studies report sensitization rates exceeding 90% for both mites [34]. Recent studies from Vietnam reported sensitization rates of 45% for *B. tropicalis* and 32% for *D. pteronyssinus* among patients with chronic respiratory diseases [35]. In South Asia, climatic heterogeneity strongly influences HDMs distribution. In humid coastal areas, all three mites frequently coexist, with sensitization rates exceeding 60% [22]. In contrast, arid inland areas, such as central India and parts of Pakistan, show a predominance of *D. farinae*, with a 20‐year study in India reporting a 47.97% sensitization rate among persistent AR patients [23, 24]. In the Middle East and West Asia, arid climates with low indoor humidity (< 50%) limit mite proliferation, resulting in lower sensitization rates [36]. However, localised humid microenvironments (particularly in coastal regions) allow for mite survival. For instance, a study from Israel showed pronounced regional variation in HDMs sensitization among AR patients: 92% in the humid coastal plains, 62% in the central mountainous areas, and 46% in the arid southern desert [37].

### 2.2 Other Indoor Allergens

Beyond HDMs, other indoor allergens also exhibit marked regional differences in sensitisation patterns. In East Asia, pet dander and cockroach allergens are common, with sensitisation rates reaching 4.02%–23.1% and 21.1%–24.5%, respectively, while mould allergens remain less prevalent (3.92%–6%) [38, 39]. In Southeast Asia, the humid climate favours cockroach sensitisation. Studies from Vietnam and Thailand report cockroach sensitisation rates ranging from 10.2% to 55.4%, whereas pet dander sensitisation is relatively low (e.g., 8.2% for cat, 2.6% for dog) [40, 41]. In South Asia, a study in northern India reported cockroach sensitisation in 18.3% of asthmatic children, notably higher than HDM sensitisation (7.8%) [42]. Pet dander sensitisation remains low in this region (3.1%) [43]. In the Middle East, despite a generally arid climate, localised humid microenvironments enable sensitisation to indoor allergens. Paediatric data from Qatar and Saudi Arabia show significant sensitisation to pet dander ranging from 14.0% to 53%, to mould from 16.0% to 18.3%, and to cockroach from 18.4% to 30.6% [44, 45, 46].

### 2.3 Outdoor Pollen Allergens

**FIGURE 2:**
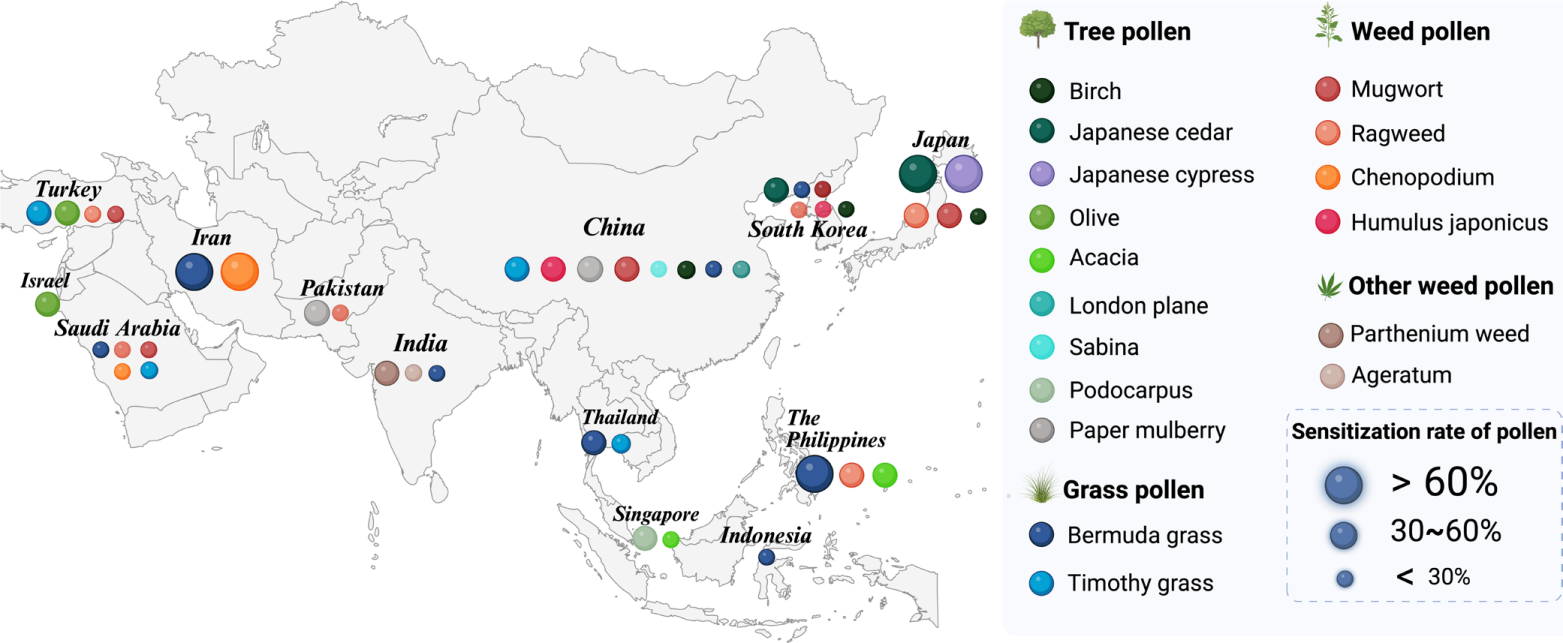
Geographic distribution of dominant sensitising pollens in Asia in the past decade.

Outdoor pollen allergens constitute a major cause of aeroallergen sensitisation in Asia, with distribution patterns largely determined by regional climate and vegetation. Table 1 and Figure 2 (Created with BioRender.com.) illustrate the predominant pollen species associated with allergic sensitisation across Asia, based on studies from the past decade.

**TABLE 1:** Pollen sensitization rates in selected Asian countries and regions.

Country	Major pollen sensitization	References
Turkey	*Phleum pratense* (Timoth grass) (18.2%–41.6%) *Olea europaea* (Olive) (37.4%–24.2%) *Ambrosia artemisiifolia* (Ragweed) (15.2%–29.5%) *Artemisia vulgaris* (Mugwort) (13.6%)	Erbay (2023) [47]
Israel	*Olea europaea* (30%–36%)	Eisenberg (2024) [37]
Saudi Arabia	*Cynodon dactylon* (Bermuda grass) (12.2%–18.1%) *Ambrosia artemisiifolia* (Ragweed) (15.3%–21.5%) *Phleum pratense* (Timoth grass) (10.8%–16.4%) *Artemisia vulgaris* (Mugwort) (3.9%–4.9%) *Chenopodium album* (Lamb's quarters) (11.4%–16.6%) *Salsola tragus* (Russian thistle) (11.7%–16.5%)	Al‐Ghamdi (2022) [48]
Iran	*Chenopodium album* (Lamb's quarters) (90.4%) *Cynodon dactylon* (Bermuda grass) (85.6%) *Salsola tragus* (Russian thistle) (87.6%)	Farrokhi (2015) [49]
Pakistan	*Broussonetia papyrifera* (Paper mulberry) (41.9%) *Cannabis sativa* (Indian hemp) (22%) *Ambrosia artemisiifolia* (Ragweed) (14%) *Taraxacum officinale* (Dandelion) (14.8%) *Soliva sessilis* (15%)	Abbas (2012) [50]
India		
Northern India	*Ageratum* (5.45%) *Holoptelia* (5.01%) *Cynodon dactylon* (Bermuda grass) (3.05%–25%) *Ricinus communis* (Castor bean) (28.33%) *Amaranthus spinosus* (28.33%–35.4%) *Parthenium hysterophorus* (Parthenium weed) (26.66%) *Eucalyptus tereticornis* (26.66%)	Laha (2022) [51] Mishra (2016) [52] Prasad (2009) [43]
Eastern India	*Cocos nucifera* (Coconut) (73.28%) *Azadirachta indica* (Neem) (57.25%) *Senna siamea* (20%)	Laha (2022) [51] Dey (2019) [53] Karmakar (2019) [54]
Southern India	*Parthenium hysterophorus* (Parthenium weed) (30.21%) *Mallotus phillippensis* (12.1%) *Prosopis juliflora* (Mesquite) (6.3%)	Laha (2022) [51] Lal (2011) [55] Rawat (2004) [56]
Central India	*Parthenium hysterophorus* (Parthenium weed) (7.7%)	Arbat (2016) [57]
Thailand	*Cynodon dactylon* (Bermuda grass) (18.9%–42.86%) *Phleum pratense* (Timoth grass) (16%) *Sorghum halepense* (Johnson grass) (12.7%–24.5%) *Cyperus* (sedge) (63%) *Urochloa mutica* (para grass) (47%)	Sangchan (2024) [58] Oncham (2018) [59] Aud‐in (2024) [60]
Singapore	*Elaeis guineensis* (Oil palm) (39.8%) *Casuarina equisetifolia* (27.7%) *Acacia auriculiformis* (Earleaf acacia) (27.7%) *Podocarpus polystachyus* (33.8%) *Kyllingia polyphylla* (Navua sedge) (25.5%)	Chew (2000) [6]
Indonesia	*Cynodon dactylon* (Bermuda grass) (3.8%)	Rengganis (2017) [61]
The Philippines	*Cynodon dactylon* (Bermuda grass) (67%) *Acacia farnesiana* (sweet acacia) (58.2%) *Ambrosia artemisiifolia* (Ragweed) (57.9%) *Sorghum halepense* (Johnson grass) (59%)	Navarro‐Locsin (2018) [62]
China		
Northern China	*Populus* spp. (Poplars) (17.2%–4.3%) *Sabina chinensi*s (24.8%) *Artemisia vulgaris* (Mugwort) (33.94%–3.1%) *Humulus japonicus* (Japanese hop) (36.2%) *Ambrosia artemisiifolia* (Ragweed) (24.4%–10.94%)	Guan (2022) [25] Wang (2022) [63] Zhang (2023) [64]
Northeastern China	*Artemisia vulgaris* (Mugwort) (33.6%) *Ambrosia artemisiifolia* (Ragweed) (29%) *Betula* spp. (Birch) (18.9%) *Corylus* spp. (Hazel) (19.9%)	Lou (2017) [65]
Eastern China	*Platanus acerifolia* (London plane tree) (17.65%) *Ambrosia artemisiifolia* (Ragweed) (1.8%–24.03%) *Artemisia vulgaris* (Mugwort) (3.4%–20.17%)	Zhao (2022) [26] Wang (2022) [63]
Central China	*Ambrosia artemisiifolia* (Ragweed) (5.3%–22.2%) *Artemisia vulgaris* (Mugwort) (9.1%–27.1%) *Betula* spp. (Birch) (21%) *Corylus* spp. (Hazel) (22%)	Wang (2024) [66] Lou (2017) [65]
Southern China	*Artemisia vulgaris* (Mugwort) (3.1%–14.9%) *Taraxacum officinale* (Dandelion) (11.7%) *Ambrosia artemisiifolia* (Ragweed) (14.3%–5.7%) *Corylus* spp. (Hazel) (7.7%) *Broussonetia papyrifera* (Paper mulberry) (38.4%) *Sorghum halepense* (Johnson grass) (10%) *Cynodon dactylon* (Bermuda grass) (17.2%) *Phleum pratense* (Timoth grass) (12.3%)	Lou (2017) [65] Zeng (2025) [67] Wu (2019) [68] Liang (2010) [69] Yong (2013) [70]
Southwestern China	*Artemisia vulgaris* (Mugwort) (54.2%–58.2%) *Ambrosia artemisiifolia* (Ragweed) (47.1%) *Betula* spp. (Birch) (24.2%) *Corylus* spp. (Hazel) (28.7%) *Phleum pratense* (Timoth grass) (28.3%–37.9%)	Wu (2021) [71] Lou (2017) [65]
Northwestern China	*Salix* spp. (Willow) (16.8%) *Humulus japonicus* (Japanese hop) (15%) *Brassica rapa* (Field mustard) (17.1%)	Wang (2024) [66]
South Korea	*Cryptomeria japonica* (Japanese cedar) (0.8%–38.1%) *Betula* spp. (Birch) (1.7%–23.9%) *Quercus* spp. (Oak) (1.1%–11.5%) *Humulus japonicus* (Japanese hop) (2.4%–16.4%) *Ambrosia artemisiifolia* (Ragweed) (0.2%–14%), *Artemisia vulgaris* (Mugwort) (1.8%–19.8%) *Cynodon dactylon* (Bermuda grass) (1.4%–7.8%) *Phleum pratense* (Timoth grass) (3.3%–4.2%) *Alnus glutinosa* (Alder) (8.8%) *Anthoxanthum odoratum* (Sweet vernal grass) (5.4%–8.4%)	Sung (2017) [72] Kim (2020) [73] Jo (2021) [74] Lee (2015) [75] Park (2019) [28]
Japan	*Cryptomeria japonica* (Japanese cedar) (51.2%–78.8%) *Chamaecyparis obtuse* (Japanese cypress) (13%–64.4%) *Artemisia vulgaris* (Mugwort) (33.1%) *Ambrosia artemisiifolia* (Ragweed) (45.6%) *Betula* spp. (Birch) (6.5%–13.8%)	Sakashita (2021) [14] Nakamura (2019) [76] Tanaka (2016) [77]

In East Asia, regional climates drive distinct pollen profiles. In northern and northwestern China, weed pollens such as mugwort and *Ambrosia artemisiifolia* (ragweed) are major allergens, with SPT positivity reaching up to 50% and 46%, respectively [78, 79]. Two pollens prominent in Korea, *Betula* spp. (birch, 10.3%) and *Alnus glutinosa* (alder, 8.8%) [72]. In Japan, Japanese cedar accounts for widespread seasonal AR, with sensitisation rates increasing from 26% to nearly 39% between 2008 and 2019 [9, 80]. Grasses such as *Phleum pratense* (Timothy grass) and *Cynodon dactylon* (Bermuda grass) are less dominant but remain clinically relevant in southern regions [79].

In Southeast Asia, the tropical climate favours sensitisation to grasses and weeds. In Thailand, SPT positivity reached 63% for *Cyperus* (sedge), 47% for *Urochloa mutica* (para grass) and 40% for Bermuda grass [60]. A study in the Philippines identified Bermuda grass (67%), *Sorghum halepense* (Johnson grass, 59%) and *Acacia farnesiana* (sweet acacia, 58%) as the leading sensitising pollens [62].

In South Asia, especially India, over 80 allergenic pollen species have been documented over the past two decades [51]. Weed pollens predominate, with *Parthenium hysterophorus* (parthenium weed) emerging as a major allergen, showing sensitisation rates ranging from 4% to 34%, and up to 40% in urban areas [57, 81]. Nationally, approximately 21.8% of respiratory allergy patients exhibit reactivity to weed pollen [82].

In the Middle East and West Asia, Mediterranean coastal areas such as Israel and Turkey report high sensitisation to *Olea europaea* (olive), while arid inland regions are dominated by weed pollens such as ragweed, *Salsola tragus* (Russian thistle), and *Chenopodium album* (lamb's quarters) [37]. A meta‐analysis from Iran revealed a sensitisation rate of up to 54% to weed pollens, followed by 40% to grasses [46]. In southwestern Saudi Arabia, reactivity to ragweed (15.3%–21%), Russian thistle (11.7%–16.5%), and lamb's quarters (11.4%–16.6%) was reported [48]. Additionally, desert trees like *Prosopis juliflora* (mesquite) and *Phoenix dactylifera* (date palm) contribute to regional allergen burdens, with sensitisation rates of 10%–18% [48].

*Broussonetia papyrifera* ( *B. papyrifera* , also known as paper mulberry) has emerged as a clinically significant allergen in multiple Asian countries. Sensitisation was reported in 38.4% of atopic individuals in Taiwan, China, and up to 41.9% in Pakistan, where airborne concentrations during peak seasons exceeded 30,000 grains/m^3^ [50, 68]. In Chengdu, a city in southwestern China, this species accounted for 58.6% of total annual airborne pollens [83]. Given its high allergenic potential [84] and widespread presence in countries such as China, Pakistan, Cambodia, Japan, South Korea, Laos, Malaysia, Myanmar, Thailand, and Vietnam [85], *B. papyrifera* may warrant consideration for inclusion in routine diagnostic panels in these regions. However, its clinical relevance in these regions requires further validation through sensitisation surveys. Standardised diagnostic reagents for *B. papyrifera* are currently lacking, and sensitisation data from many parts of its distribution range remain unavailable. Developing regionally validated, standardised extracts will be essential for accurate diagnosis and potential immunotherapeutic development.

### 2.4 Molecular Allergen Components Identified in Asian Pollen Species

Although Table 1 outlines the predominant pollen species reported across Asia, component‐resolved diagnostic (CRD) data remain limited in most regions. In China, CRD studies have identified Art v 1 (mugwort) and Amb a 1 (ragweed) as major allergens [86], highlighting significant cross‐reactivity between weed pollens and food allergens via profilins and lipid transfer proteins (LTPs). Bro p 3, an nsLTP1, was recently identified as the first major allergen of paper mulberry, a regionally important pollen source [84]. In Japan, Japanese cedar pollen allergy is characterised by sensitisation to Cry j 1 and Cry j 2, which have been incorporated into both diagnostics and commercial immunotherapy preparations [87]. In Korea, Bet v 1 and Bet v 2 are dominant components in birch sensitisation, particularly in children with oral allergy syndrome [88]; Hum j 6, a defensin‐like protein, was also identified as the major allergen of *Humulus japonicus* [89]. In Iran, CRD studies on *Chenopodium album* (Lamb's quarters) revealed Che a 1 (Ole e 1–like), Che a 2 (profilin), and Che a 3 (polcalcin) as key IgE‐binding components [90].

## 3 Factors Influencing Aeroallergen Sensitization in Asia

### 3.1 Geographical and Climatic Influences

Asia's extensive geographic and climatic diversity significantly influences regional aeroallergen exposure and sensitisation patterns. Humid tropical and subtropical climates, notably in Southeast Asia (e.g., Singapore, Vietnam, southern China), favour proliferation of HDMs, resulting in sensitisation rates exceeding 75% [21, 62, 63, 91, 92]. Conversely, arid regions like Saudi Arabia report HDMs sensitisation rates of up to 15% [48]. Mould allergen sensitisation similarly correlates with humidity, with prevalence reaching 70% among adults in the Philippines [62]. Pollen sensitisation exhibits a different pattern; it is relatively uncommon in equatorial zones but increases with latitude. Climate change further exacerbates these burdens by extending pollen seasons, increasing pollen yield, and enhancing allergenicity [93, 94, 95, 96, 97]. Altered precipitation patterns can also modulate allergen dispersion. While rainfall typically suppresses airborne pollen, it may simultaneously rupture pollen grains and promote allergen release, leading to episodes of ‘thunderstorm asthma’. Such events, characterised by sudden asthma exacerbations triggered by thunderstorm‐driven dispersal of allergenic particles, have been reported not only in China [98] but also in other regions including Australia [99, 100].

### 3.2 Urbanisation and Lifestyle

Rapid urbanisation and associated lifestyle changes have markedly increased the prevalence of allergic diseases across Asian urban centres. According to the ‘hygiene hypothesis’, reduced microbial exposure in early life—common in urban environments—may impair the development of immune tolerance [101]. Supporting this, studies have shown that children raised on farms, exposed to raw milk and diverse microbial stimuli, exhibit enhanced regulatory T cell responses and a lower risk of asthma and allergic diseases [102].

In urban areas, widespread use of air‐conditioning further influences indoor allergen dynamics [103]. While air‐conditioning typically reduces indoor humidity and potentially inhibits the proliferation of allergens such as HDMs, inadequate maintenance and infrequent cleaning of air‐conditioning systems can lead to accumulation and recirculation of allergens within indoor spaces [104, 105], thereby exacerbating allergen exposure and related respiratory symptoms [106, 107]. As a result, urban children exhibit higher sensitisation rates to HDMs compared with rural counterparts (e.g., 71.9% vs. 45.9% in Taiwan, China) [108]. Moreover, increased pet ownership in urban areas (e.g., Tokyo, Shanghai) contributes to higher pet dander sensitisation [101, 109, 110]. Notably, the immunological impact of pet exposure appears to be age‐dependent: early‐life exposure may promote immune tolerance, whereas exposure during adulthood may increase allergy risk [111].

However, the effects of urbanisation on allergen sensitisation vary significantly between countries due to distinct environmental, infrastructural, and policy‐related factors. In China, rapid urban expansion has led to increased indoor allergen exposure, driven by high‐rise housing, suboptimal ventilation, and increased reliance on air‐conditioning. In contrast, Japan faces a unique pollen‐related challenge due to post‐war afforestation policies, which led to the mass planting of Japanese cedar and cypress in urban and suburban areas. These species now contribute substantially to the seasonal pollen load in Japan, with sensitisation rates to cedar pollen reaching 60% among young adults in metropolitan areas [14]. Furthermore, Japan's national pollen surveillance and forecasting system likely increases diagnostic uptake and public awareness, further shaping reported sensitisation trends.

While urbanisation is generally associated with higher sensitisation rates, this association is influenced by various confounding factors such as socioeconomic status, healthcare access, and diagnostic capabilities. In urban centres, the increased availability of allergy testing—such as skin prick tests (SPT) and serum‐specific IgE assays—may contribute to higher reported sensitisation rates, even without a corresponding rise in clinical allergy cases [112].

### 3.3 Air Pollution

Air pollution has emerged as a critical environmental factor influencing aeroallergen sensitisation patterns in Asia. Rapid industrialisation has rendered the region among the most polluted globally, with elevated levels of particulate matter (PM_2.5_, PM_10_), nitrogen dioxide (NO_2_), and ozone (O_3_) significantly altering both allergen exposure and allergenicity [113]. Pollution affects aeroallergens through multiple interconnected mechanisms.

First, elevated pollutant levels, such as PM_2.5_, NO_2_, and O_3_ increase the concentration of airborne allergens by promoting the release of allergenic particles from various sources, including plant pollen and dust mites [113]. These pollutants can also interact with allergens, increasing their stability and persistence in the environment, thus prolonging exposure and enhancing allergenic potential.

Second, oxidative stress induced by pollutants plays a central role in altering the structure of allergenic proteins. Chemical processes such as nitration, oxidation, and oligomerisation have been shown to modify the molecular structure of allergens, increasing their IgE‐binding capacity and enhancing the immune response [114]. For instance, exposure to NO_2_ and SO_2_ in polluted environments has been linked to changes in the structure of pollen proteins, such as those from birch and *Acer negundo* , which increase their allergenic potential [115, 116].

Third, pollution‐induced environmental stress on plants stimulates the overproduction of allergenic proteins [117, 118]. For example, Japanese cedar pollen from urban areas in Japan exhibits greater pollutant adsorption compared to rural samples, leading to stronger inflammatory responses [119]. Airborne particulates may further facilitate allergen delivery by transporting allergen–protein complexes deeper into the respiratory tract and promoting epithelial inflammation. Collectively, these pollution‐related mechanisms substantially exacerbate respiratory allergic diseases, notably in heavily affected countries such as India, China, and Indonesia, which together account for a significant share of the global asthma burden [120].

### 3.4 Sex, Age, and Genetic Determinants

Biological factors—including sex, age, and genetic predisposition—significantly influence the heterogeneity of aeroallergen sensitisation across Asia. Understanding these intrinsic influences is critical to interpreting the diverse allergic disease patterns observed in different populations. Male predominance in allergic sensitisation during childhood has been consistently reported across various populations. For example, studies from Europe, including a longitudinal Finnish cohort, demonstrated that males exhibited higher rates of sensitisation and polysensitisation [120, 121]. Similar trends have been confirmed in Asian populations [122, 123]. A large retrospective study in over 4000 patients from South Korea revealed that males were generally more frequently sensitised than females except for fungal allergens [122]. A recent Japanese birth cohort study found that higher serum testosterone levels in male children were associated with an increased risk of developing allergic diseases [123]. Sex hormones likely modulate immune responses through receptor‐mediated effects on cytokine production and lymphocyte activity [124, 125]. Age also plays a crucial role, and the trajectory of sensitisation varies by allergen type [126]. For instance, HDMs sensitisation shows a marked age‐dependent increase in tropical regions. In Singapore, HDMs sensitisation rates were reported to rise from 84.5% in children aged 3–5 years to 97.3% in those older than 14 years [127]. This pattern reflects both cumulative environmental exposure and age‐related maturation of the immune system.

Genetic studies conducted in Asian populations have increasingly identified specific gene variants associated with allergic susceptibility. For instance, polymorphisms in *ADAM33* have been linked to asthma risk in Japanese children [128]. Genome‐wide association studies (GWAS) have further revealed several loci—including CD28, IL7R, STAT6, and HLA variants—that contribute to mono‐ and polysensitization [129]. In a cohort from Taiwan, China, single nucleotide polymorphisms (SNPs) in CD28, 11q23.2, and HLA class II genes were strongly correlated with elevated total serum IgE levels and enhanced allergen reactivity [130]. Recent studies from mainland China have provided additional support; Zhao et al. [131] identified specific HLA alleles, such as HLA‐DRB108:03:02* and HLA‐DQB106:01:01*, associated with HDMs sensitive AR. Furthermore, interactions among key genes involved in effector T‐cell pathways have been shown to increase susceptibility to AR [132], and polymorphisms in MRPL4 and TNF‐α genes were also found to contribute to allergic risk [133]. Notably, a recent genome‐wide analysis revealed that positive selection signatures are enriched at allergy‐associated loci [134], suggesting that evolutionary pressures may have shaped the genetic architecture of allergic diseases in Asian populations.

## 4 Prevention and Management Strategies

Understanding region‐specific allergen sensitisation patterns is crucial for accurate diagnosis and effective management of allergic diseases in Asia. Environmental control remains a foundational strategy, including the use of impermeable mattress covers, removal of indoor pets, enhanced ventilation, and wearing masks during high pollen seasons, among others [135]. However, due to the ubiquity of allergens—particularly in polysensitised individuals—complete avoidance is often impractical.

Pharmacotherapy continues to be the primary method for symptom control. Most Asian countries follow global guidelines such as ARIA (Allergic Rhinitis and its Impact on Asthma) and GINA (Global Initiative for Asthma), employing H1‐antihistamines and intranasal corticosteroids for AR, as well as inhaled corticosteroids (ICS) with or without long‐acting β_2_‐agonists (LABAs) for asthma. Leukotriene receptor antagonists and bronchodilators are also widely used [136, 137]. In recent years, biologic therapies—such as omalizumab (anti‐IgE), mepolizumab and benralizumab (anti‐IL‐5/IL‐5R), and dupilumab (anti‐IL‐4/13)—have been introduced for severe or refractory cases. A recent Phase 3 trial in China demonstrated the efficacy of Stapokibart (anti‐IL‐4/IL‐13) in improving seasonal AR symptoms [138]. However, pharmacologic treatments primarily offer symptomatic and anti‐inflammatory relief, and symptoms often recur after therapy cessation.

AIT, administered via subcutaneous (SCIT) or sublingual (SLIT) routes, remains the only disease‐modifying intervention, capable of inducing sustained immune tolerance by gradually modulating the immune response from a Th2 to Th1 [124]. Increasing evidence supports the efficacy and safety of AIT for AR and AA, with studies demonstrating both short‐ and long‐term effectiveness [139, 140, 141, 142]. However, AIT uptake across Asia remains low, with utilisation rates often below 10% in many regions [143], hindered by factors such as limited availability of standardised allergen extracts, high treatment costs, regulatory constraints, and low patient and physician awareness [144] (Table 2 shows the use of AIT in selected Asian countries).

**TABLE 2:** Allergen immunotherapy use in selected Asian countries.

Country	SCIT	SLIT
Mainland China	Two standardised HDMs allergen extracts approved by NMPA. Novo Helisen‐Depot (Allergopharma, Germany), Alutard‐SQ (ALK‐Abelló, Denmark) [145] In 2012, nine types of non‐standardised crude allergen extracts approved for limited hospital use	*D. farinae* drops (Wolwo Bio‐Pharmaceutical, China) approved in 2006 [145] HDMs tablet (ACARIZAX, ALK‐Abelló, Denmark) introduced in 2023 under special program Mugwort SLIT drops (Wolwo Bio‐Pharmaceutical, China) approved in 2021 No widely approved SLIT for other pollens or animal dander
Japan	SCIT for Japanese cedar pollen and HDMs historically practiced but declined after SLIT approval SCIT for other pollens or pet dander is rare	SLIT drops for *Japanese cedar* pollen (Cedartolen, Torii, Japan) has been covered by national health insurance since 2014 [146] SLIT tablets for *Japanese cedar* pollen (Cedarcure, Torii, Japan) was approved in 2018 [80] SLIT tablets for HDMs (Miticure, Torii; Actair, Shionogi) approved and reimbursed
South Korea	SCIT for HDMs is common, mostly imported (Tyrosine S, Allergy Therapeutics, UK; Novo‐Helisen Depot, Allergovit, Germany) [147] SCIT for some pollens (birch, ragweed, mugwort) and moulds or animal dander via imported [147]	SLIT for HDMs available (Lofarma, Stallergenes, ALK) [148] No officially approved SLIT for pollens
India	SCIT widely practiced using aqueous “native” extracts for HDMs, local weeds (e.g., *Parthenium hysterophorus* ) grasses, tree pollens, cockroach, and fungi [51]	SLIT is recognised but limited use No officially approved SLIT tablets Mostly off‐label aqueous drops [51]
Southeast Asia (e.g., Singapore, Malaysia, Thailand)	SCIT available via imported products in Malaysia (named‐patient basis) [149] Urban centers (Singapore, Bangkok, Kuala Lumpur) offer SCIT for moderate–severe cases	SLIT drops for HDMs available in some clinics for children Singapore and Thailand use SLIT for HDMs and some pollens, though products may be off label [150]

Abbreviation: NMPA, National Medical Products Administration.

In China, AIT has been practised for over 60 years, initially using non‐standardised, in‐house crude extracts [151]. A real‐world study involving 246 patients with AR (with or without asthma) showed that 96.7% reported symptom improvement after SCIT using crude extracts [152]. For more than two decades, standardised HDM extracts have been available for SCIT, with three currently approved products: Novo Helisen‐Depot (Allergopharma, Germany), Alutard‐SQ (ALK‐Abelló, Denmark) for SCIT, and Challergen‐*Dermatophagoides farinae* Drops (Wolwo Bio‐Pharmaceutical, China) for SLIT [145]. A head‐to‐head comparison of *Der p* (Alutard SQ) and *Der p/Der f* (Novo Helisen‐Depot) showed comparable efficacy and safety [153]. In 2021, mugwort SLIT drops were approved for seasonal AR patients in China, supported by a Phase 3 randomised controlled trial showing significant symptom reduction during peak pollen period [154].

In India, AIT is primarily administered via SCIT using crude, non‐standardised “native” allergen extracts derived from HDMs, moulds, and regionally prevalent pollens [51]. A retrospective analysis found symptom improvement in only 42% of polysensitised AR patients, possibly due to variable protease activity in crude extracts affecting therapeutic consistency [155]. Standardised and quality‐controlled commercial products are largely unavailable, and SLIT is limited due to regulatory and cost barriers.

In Southeast Asia, access to AIT is mostly limited to tertiary care centers in urban areas, constrained by economic and regulatory barriers [149]. Thailand primarily uses imported allergen extracts, mainly for HDM allergy, with no commercially licensed standardised pollen SLIT or SCIT products available locally. In contrast, Japan and South Korea have developed well‐established AIT programs supported by insurance reimbursement and robust clinical trial evidence [80, 146]. In Japan, both SCIT and SLIT for HDMs or Japanese cedar pollen are nationally approved and widely available [80]. A 3‐year real‐world study in 768 Japanese patients withHDM‐induced AR treated with standardised SLIT tablets (Miticure, Torii Pharmaceutical Co. Ltd.) demonstrated sustained symptom improvement: 67.3% of patients reported improved quality of life at 6 months, which increased to over 90% at 2 years. Japanese cedar pollen AIT, uniquely available in Japan, includes both SLIT drops (Cedartolen) and tablets (Cedarcure), widely reimbursed by the national healthcare system. However, no standardised products are commercially available for other pollens like ragweed or birch. In South Korea, it primarily offers SCIT for pollens—most of which are imported from Europe—with SLIT currently limited to HDMs [147]. A retrospective study of 117 adults with AA found that long‐term AIT can reduce ICS use and improve asthma control [156]. Notably, local allergens such as oak and Japanese hop lack registered AIT products, significantly limiting therapeutic options.

Overall, both SCIT and SLIT have shown good efficacy in symptom control for AR or AA and are recommended as first‐line treatments by global guidelines such as those from the European Academy of Allergy and Clinical Immunology (EAACI) and ARIA, due to their long‐term benefits [157]. However, regional disparities in AIT availability and implementation across Asia reflect broader differences in healthcare infrastructure, regulatory approval processes, and allergen extract standardisation, all of which may influence the effectiveness of AIT. Notably, several regionally important pollen allergens still lack standardised AIT products across Asia, including paper mulberry (China, Pakistan), Parthenium weed, mesquite tree, and Bermuda grass (India), oak and Japanese hop (South Korea), and multiple local weeds and grasses in Southeast Asia. Addressing these gaps is essential to improving region‐specific allergy care.

Beyond pharmacological treatments, improving treatment adherence and patient outcomes depends significantly on patient education. Public health campaigns and clinician training programmes play a pivotal role in raising awareness about allergic diseases, contributing to more effective management strategies [158]. However, challenges such as lack of awareness and patient education programmes in many regions persist and must be addressed.

## 5 Strategies to Improve Allergy Diagnosis and Management in Asia

Understanding the region‐specific landscape of allergen sensitisation is essential to improving allergy diagnosis and treatment across Asia. Despite the availability of pharmacological and biologic therapies, AIT remains underutilised due to multiple barriers, including limited access to standardised extracts, high costs, regulatory inconsistencies, and uneven clinical expertise. To address these challenges, a comprehensive, multifaceted strategy is essential.

First, establishing publicly accessible, regionally coordinated pollen and fungal spore monitoring networks is critical. Real‐time environmental data would improve diagnosis, guide preventive behaviours, and support anticipatory public health responses. China's national atmospheric pollen surveillance system, launched in 2019, may serve as a model for broader Asia‐wide initiatives [159]. An integrated Asian monitoring platform could consolidate resources, collaborate with meteorological departments, and promote standardised data collection. Some European networks, such as EAN (European Aeroallergen Network) and Germany's PID (Pollen Information Service) system, have shown the potential for regional coordination and public access to data [160]. With advances in artificial intelligence (AI), automated image recognition and deep learning technologies may further enhance the accuracy and efficiency of pollen identification. To facilitate the development of a pan‐Asian network, international collaborative frameworks should be leveraged. The World Health Organisation (WHO) South‐East Asia and Western Pacific Regional Offices, along with Association of Southeast Asian Nations (ASEAN) health initiatives, offer strategic platforms for cross‐border coordination, resource sharing, and data harmonisation. Regional allergy organisations such as the Asia Pacific Association of Allergy, Asthma and Clinical Immunology (APAAACI) and World Allergy Organisation (WAO) could contribute to the establishment of standardised monitoring protocols, quality control, and governance mechanisms. While initial investments in infrastructure and workforce training may pose financial and logistical challenges, the long‐term benefits–including improved diagnostic accuracy, reduced misdiagnosis, enhanced public health preparedness, and optimised patient care–are likely to outweigh the costs.

Second, harmonising the quality and regulation of allergen extracts across Asia is urgently needed to ensure reliable diagnosis and consistent immunotherapy outcomes. Drawing from the success of the CREATE project in Europe—which developed certified reference materials and validated analytical methods [161]—Asia would benefit from a regional initiative to standardise diagnostic reagents and immunotherapeutic formulations. Such harmonisation would enable cross‐border comparability, support multicentre clinical trials, and facilitate regulatory convergence, helping establish regionally tailored standards for allergen therapy.

Third, bridging the gap between the clinical potential and real‐world application of AIT requires stronger policy support. Key priorities include the establishment of national reimbursement systems, regulatory facilitation for standardised extracts, and structured clinician training embedded within continuing medical education. These efforts will help expand the trained workforce and promote evidence‐based AIT across the region. Governments and international organisations should collaborate to establish robust frameworks for the equitable distribution of AIT resources and the alignment of regional regulatory practices.

In parallel, greater investment in regional basic and translational research is essential. Priority areas include population‐specific immunogenetic profiling, component‐resolved diagnostics, and long‐term outcome studies of AIT in Asian cohorts. Multinational collaboration, harmonised study protocols, and data‐sharing platforms will help generate robust evidence and inform locally adapted clinical guidelines. Strengthening research partnerships, especially in underserved regions, will further promote localised treatment strategies and improve overall health outcomes.

## 6 Limitations

While this review provides a comprehensive overview of aeroallergen sensitisation across Asia, several limitations should be acknowledged. First, the heterogeneity of diagnostic methods introduces significant potential for bias. Although SPT and serum‐specific IgE assays are the most commonly used tools for allergen sensitisation assessment [162], their application varies widely in terms of allergen panels, diagnostic thresholds, and level of extract standardisation. Differences in the quality and origin of allergen extracts—ranging from internationally validated commercial products to locally compounded crude formulations—may significantly affect sensitivity estimates and inter‐study comparability. For instance, in countries such as China and India, pollen and dander extracts are often produced by individual institutions without uniform quality control, further complicating cross‐regional interpretations [141].

Second, although efforts were made to include recent and representative data from across Asia, regional disparities remain. Data from Central and parts of Southeast Asia are particularly scarce, which may result in a disproportionate focus on East Asian countries. Additionally, some included studies relied on smaller sample sizes or targeted specific age groups (e.g., children or urban adults), which may not reflect general population trends.

Third, while our search strategy focused on the most recent decade (2015–2025), some earlier publications were retained to provide baseline data for under‐represented regions. The inclusion of older studies may introduce variability due to changes in diagnostic practices or environmental exposures over time.

Finally, the methodological heterogeneity mentioned above precluded the feasibility of constructing a comparative summary table across all studies. Moreover, due to the narrative scope of this review, we did not conduct advanced quantitative analyses such as meta‐analysis or Mendelian randomisation. Such approaches could help clarify causal relationships and strengthen the robustness of conclusions drawn. Therefore, future research should consider employing these methodologies to provide more definitive and reliable evidence. Nonetheless, this review emphasises the urgent need for harmonised diagnostic protocols and regionally coordinated allergen surveillance efforts to improve the interpretability and applicability of sensitisation data in clinical and public health contexts.

## 7 Conclusions

This review highlights the substantial regional heterogeneity in aeroallergen sensitisation across Asia, shaped by diverse climatic, geographic, environmental, and host factors. While HDMs remain the dominant indoor allergens, pollen sensitisation varies widely by region and season. Despite progress in pharmacologic and biologic treatments, AIT remains the only disease‐modifying option. Yet, its implementation across Asia remains limited due to challenges in standardisation, access, regulation, and clinical capacity. To move forward, strategic priorities include establishing coordinated pollen monitoring systems, harmonising allergen extract standards, enhancing clinician training, and increasing investment in region‐specific research. As environmental and social transformations continue to reshape exposure patterns, implementing a precision medicine framework tailored to Asia's diversity will be key. Cross‐sector collaboration among policymakers, researchers, and clinicians is essential to reduce the burden of allergic diseases and improve long‐term outcomes for affected populations.

## Author Contributions

P.H.L. and J.M. conceived the idea and led the write‐up. X.Y. and X.Z. wrote the initial draft. All authors contributed to obtaining data. All authors revised and approved the final manuscript.

## Conflicts of Interest

The authors declare no conflicts of interest.

## Data Availability

The authors have nothing to report.

## Appendix A

We conducted a narrative review by performing a comprehensive literature search across PubMed, Web of Science, Scopus, and Google Scholar for studies published between January 2010 and May 2025, with a focus on research from 2015 onward. The search was based on a combination of population terms (e.g., ‘Asia’, ‘China’, ‘India’), aeroallergen exposure terms (e.g., ‘pollen’, ‘dust mite’, ‘cockroach’), and outcome terms (e.g., ‘sensitization’, ‘SPT’, ‘specific IgE’). Boolean operators (AND/OR) were used to combine these terms in each database, with slight adjustments to accommodate each platform's specific search syntax.

We included original studies on sensitisation to indoor or outdoor aeroallergens in Asian populations using skin prick tests, specific IgE, or component‐resolved diagnostics, involving both paediatric and adult populations, and published in English. We excluded review articles, case reports, conference abstracts without primary data, studies lacking clear diagnostic methods, and studies conducted outside Asia. Duplicates across databases were removed, and titles, abstracts, and full texts were screened by two independent reviewers, with discrepancies resolved through discussion.
